# Heart rate variability and inflammatory markers in neonates with hypoxic‐ischemic encephalopathy

**DOI:** 10.14814/phy2.14110

**Published:** 2019-08-08

**Authors:** Daphna Yasova Barbeau, Charlene Krueger, Melissa Huene, Nicole Copenhaver, Jeffrey Bennett, Michael Weaver, Michael D. Weiss

**Affiliations:** ^1^ Department of Pediatrics University of Florida Gainesville Florida; ^2^ College of Nursing University of Florida Gainesville Florida; ^3^ Department of Radiology University of Arizona Tuscon Arizona; ^4^ Department of Statistics University of Florida Gainesville Florida; ^5^ Department of Biostatistics University of Florida Gainesville Florida

**Keywords:** Biomarkers, heart rate variability, HIE, HRV, hypoxic ischemic encephalopathy, neonatal asphyxia

## Abstract

To examine heart rate variability (HRV) and inflammatory markers as predictors for neurological injury in neonates undergoing therapeutic hypothermia for hypoxic‐ischemic encephalopathy (HIE). We hypothesized that HRV would differentiate between infants with no/mild injury and infants with moderate/severe injury observed on MRI. Because HRV can be associated with the inflammatory cascade, cytokine concentrations were compared with the severity of brain injury indicated by MRI. Further, we studied the effect of temperature, sex, and mechanical ventilation on HRV. HRV was prospectively collected on neonates with HIE using spectral analysis for low and high frequency components (n = 16). A subset (n = 10) of neonates had serum available for inflammatory cytokine analysis obtained during cooling. Neonates were stratified into no/mild or moderate/severe injury based on MRI obtained after rewarming. Differences in HRV were identified; lower low frequency power predicted more injury on MRI. Additionally, in neonates with HIE after cooling procedure, HRV differed by gender. Elevated RANTES (CCL5) and decreased GM‐CSF (Granulocyte‐macrophage colony‐stimulating factor) at 96 hours predicted less severe injury. In this small study, HRV differs between no/mild and moderate/severe injury in neonates with HIE. With further study, this may aid the clinician in real‐time decision making. HRV differs by gender. Finally, inflammatory biomarkers may help elucidate the pathophysiology of HIE.

## Introduction

Hypoxic‐ischemic encephalopathy (HIE) is a serious birth complication occurring in approximately 3–5 out of 1000 full‐term infants (MacDonald et al. 1980; Low et al. 1997). Studies of therapeutic hypothermia (TH), the current standard therapy, showed that TH decreased mortality with a relative risk of 0.77 and confers a risk difference of −16% in infants with HIE (Jacobs et al. 2013). Also, TH improves the neurological and neurodevelopmental outcome of up to 53% of treated infants and provides a 60% decrease in relative risk of cerebral palsy (Jacobs et al. 2013; Azzopardi et al. 2014). Despite improvement in outcomes since the advancement of TH, the bedside clinician is still not able to identify which neonates will respond to TH and which will not. Therefore, a marker that helps predict response to treatment would be beneficial.

Current monitoring and evaluation of HIE, outcome prediction, and efficacy of TH rely on a combination of a neurological exam, ultrasound, amplitude electroencephalography (aEEG), and magnetic resonance imaging (MRI) (Leijser et al. 2007; van Laerhoven et al. 2013). The requisite 45‐min MRI, the gold standard for identifying hypoxic‐ischemic brain injury, requires the neonate to be transported. Travel to the MRI suite is inappropriate for unstable neonates and in addition, MRI is used for diagnosis of HIE not predicting the development of HIE. As a result, limits the utility of MRI as a decision‐making tool before or during hypothermia. Both the neurologic examination and the aEEG can be impacted by medications such as sedatives and by hypothermia itself (Gunn et al. 2008; Thoresen et al. 2010). Neurologic exam does tend to correlate with severity of HIE, though is not predictive for every infant. While ultrasound can be performed at the bedside, ultrasound is most useful in identifying the cystic form of white matter injury, a late finding, and is not sensitive enough to evaluate even diffuse white matter injury (Miller et al. 2005; Martin et al. 2014). Therefore, the development of a new, rapid, and reliable prognostic test is essential for clinicians who need to make critical therapeutic decisions during hypothermia.

In addition to current physiologic bedside monitoring, heart rate variability (HRV) may be a potential biomarker for neonates with HIE. HRV is the measurement of intervals between heartbeats and is regulated by the sympathetic and parasympathetic branches of the autonomic nervous system. Changes in HRV can be an indicator of neuroinflammation and neonatal sepsis (Rajendra Acharya et al. 2006; Fairchild and O'Shea 2010). Research has shown that a reduction in HRV predicts increased morbidity and mortality in a number of pathological conditions (Tsuji et al. 1996). The utility of HRV is increasingly being studied in neonates.

Though small, an increasing body of evidence indicates that the autonomic nervous system may also regulate or affect the inflammatory response. While not all injury from hypoxic‐ischemic encephalopathy is due to inflammation, ischemia is thought to elicit an inflammatory cascade that involves cytokine release, exhaustion of high‐energy phosphates, and accumulation of cytotoxic cerebral edema (Martin et al. 2014; Riljak et al. 2016). In neonates, inflammatory cytokines such as IL‐1, IL‐1B, and IL‐6 correlate with neurodevelopmental abnormalities (Ramaswamy et al. 2009; Massaro et al. 2013; Lv et al. 2015; Orrock et al. 2016; Satriano et al. 2017). Although the markers of inflammation can be measured, sample processing requires additional time and the inflammatory markers only provide a snapshot of the rapidly changing milieu of post‐HIE injury. Because HRV appears to be associated with the autonomic nervous system and inflammation, HRV may provide the bedside clinician with a powerful tool to predict outcomes and glean information about the individual neonate's inflammatory response to hypoxic‐ischemic injury.

The objective of this study was to examine the relationship between measures of HRV and MRI results in infants undergoing TH for HIE. We hypothesized that when evaluating the frequency powers of HRV, increased low frequency power, reflecting higher sympathetic activity, would be associated with moderate/severe MRI brain injury because the sympathetic nervous system mediates a pro‐inflammatory state during physical stress. Additionally, this study aims to quantify the serum concentrations of inflammatory cytokines over time after hypoxic‐ischemic injury and compare these results to the extent of brain injury, as detected by MRI. While HRV has been previously studied in HIE, this study adds to the body of literature by evaluating the effect of sex, pressor use, and mechanical ventilation on HRV in neonates undergoing TH.

## Materials and Methods

### Patient populations

The University of Florida Institutional Review Board approved this study. Subjects (*n* = 16) were enrolled at the University of Florida Health Shands Hospital after parents provided informed consent. Patients with HIE who were eligible for hypothermia therapy were recruited. Entry criteria for hypothermia included a gestational age of 35 weeks or greater, a birth weight of 1.8 kg or greater, and less than or equal to 6 h of age. The neonates had evidence of encephalopathy as defined by seizures or abnormalities on a modified Sarnat exam (level of consciousness, spontaneous activity, posture, and tone, primitive reflexes including suck and Moro, and autonomic system findings including pupils, heart rate, and respirations)(Sarnat and Sarnat 1976). Evidence of hypoxic‐ischemic injury was defined as (1) a pH of 7.0 or less and/or a base deficit of greater than 16, or (2) a pH between 7.01 and 7.15 and/or a base deficit between 10 and 15.9, or (3) no blood gas available and an acute perinatal event (cord prolapse, fetal bradycardia, uterine rupture).

### Heart rate variability evaluation

Measures of HRV were obtained from a 300 sec epoch of the electrocardiogram (ECG) signal and measured twice: once during cooling (33.5°C), between 6 and 72 h of life, and once after rewarming (>36.5°C). Following communication with the bedside nurse, the infant's ECG leads were transferred to a roving cardiac monitor for measurement while the infant remained in their assigned crib. The 300 sec epoch for each measurement began once the infant had achieved a quiet‐alert period of activity. Evaluation of quiet‐alert criteria included movement (gross motor and fine motor) and respiration. Behavioral criteria were used to determine if subjects were in a quiet state were (1) irregular respirations, (2) the absence of gross motor body movement, and (3) closed eyes (Holditch‐Davis et al. 2004). If the quiet state criteria were not met, the test session was terminated.

To evaluate the effect of mechanical ventilation on HRV, a baseline recording was captured. Then, the ventilator rate was decreased to zero for a period of seconds. A physician stood at the bedside during the rate decrease and increased the rate immediately if the saturations dropped. Neonates were only selected to have the ventilator rate decreased if they were mechanically ventilated with rates of 20 or less and were close to extubation. If the neonates had pulmonary hypertension or were on a rate greater than 20, they did not have the ventilator rate turned off for the second interval. Thusly, there are not measurements for every infant on and off the ventilator.

### Heart rate variability processing

Spectral analysis of the series of R‐R intervals (heart periods) was performed offline using a Lomb procedure. The Lomb procedure was chosen because it does not require the interval between consecutive samples to be uniform. (Lomb 1976). ECG electrodes were attached to the subject in the standard three‐lead manner. Data was transferred from the monitor (model #1092A, Agilent, Santa, CA) to a laptop (Inspiron 8100, Dell©, Round Rock, TX) via an RS232 interface. Heart periods taken from the ECG signal were sampled at equal intervals for 45 sec at a rate of 500 Hz. MATLAB (MathWorks^®^, Natick, MA) was used to filter the signal by removing noise powers and baseline wander. Next, the filtered signal was passed through a QRS detection algorithm (MATLAB), resulting in a time series of R‐R intervals.

Frequencies examined included the total spectrum, high frequency (HF), low frequency (LF) and ratio of low‐to‐high frequencies (LF/HF). The total spectrum (0.04–1.0 Hz) was examined except for very low frequencies (0.0–0.04 Hz) because these powers are typically produced by slow‐trend artifacts (or noise). The high frequency power (0.30–1.00 Hz) is felt to occur in response to respirations and is controlled by the parasympathetic nervous system (commonly referred to as parasympathetic tone). The low‐frequency power (0.04–0.20 Hz), reflects the sympathetic tone. Further, the ratio of low‐to‐high frequency power (LF/HF; 0.04–0.20/0.30–1.00 Hz) reflects a combination of sympathetic and parasympathetic tone (Chatow et al. 1995; Doyle et al. 2009).

### Serum sample collection

Blood samples from neonates with HIE (*n* = 10) were prospectively collected from 10 of the 16 patients at four time points, (0–6, 24, 48, and 96 h of life), when possible. For the remaining four infants, no blood was collected at any of the time points. Initial blood lactates and pH were obtained within the first 6 h. For infants delivered outside of our facility, we used initial gases reported by the outside hospital. These time points were chosen to establish a baseline at the initiation of hypothermia, during hypothermia, and 24 h after rewarming to examine the changes in the inflammatory cytokines over time. Samples were collected at the same time that other clinical samples were collected and managed by standardized protocol, including prompt freezing to prevent breakdown. Blood (1 mL) was collected using a 3.5 mL serum separator tube (SST Plus Blood Collection Tube, BD Vacutainer^®^, Franklin Lakes, NJ). Samples were allowed to clot upright at room temperature for 30 min in the processing lab (45 ± 15 min from time of collection), then centrifuged at 1200 RCF (g) at room temperature for 15 min in a fixed angle centrifuge rotor. Then, serum was transferred using a disposable transfer pipette into 2 mL cryovials with red cap inserts (USA Scientific^®^, Ocala, Florida). The samples were placed in biorespository at −80°C until processing.

### Cytokine analysis

Cytokines were quantified with ultrasensitive ELISA technology according to manufacturer instructions (Kit Bio‐Plex Pro^™^ Human Cytokine Standards Group I 21–Plex, Kit Bio‐Plex Pro^™^ Human Cytokine Standards Group II 27–Plex, Bio‐Rad, Hercules, CA). Analysis included Cytokines: IL‐1*α*2, IL‐2R*α*, IL‐3, IL‐12p40, IL‐16, IL‐18, IL‐1*β*, IL‐1ra, IL‐2, IL‐4, IL‐5, IL‐6, IL‐7, IL‐8, IL‐9, IL‐10, IL‐12p70, IL‐13, IL‐15, IL‐17; Chemokines: CTACK, GRO‐*α*, LIF, MCP‐3, MIF, MIG, TRAIL, IP‐10, MCP‐1, MIP‐1 *α*, MIP‐1*β*, INF‐*γ*; Growth factors: HGF, M‐CSF, GM‐CSF, *β*‐NGF, SCF, SCGF‐*β*, SDF‐1*α*, TNF‐ *β*, Eotaxin, Basic FGF, G‐CSF, GM‐CSF, PDGF‐PP, RANTES, TNF‐*α*, VEGF.

### MRI scoring

MRI was performed between 4 and 12 days of age when the individual subjects were stable enough for transport. All subjects were scanned on the same 3T scanner (Verio; Siemens©, Erlangen, Germany) with a 32‐channel head coil. Analysis focused on the T1‐weighted, T2‐weighted, and diffusion weighted imaging (DWI) abnormalities. A single subspecialty board‐certified, blinded neuroradiologist with 10 years of experience in neonatal imaging used the Barkovich scoring system to interpret the MRI images. The Barkovich scoring system scores injury in different brain regions using a scale with increasing values representing worsening injury. Individual brain regions scored included the basal ganglia and thalamus (0–4), the cortex/white matter or watershed score (0–5), and a combined basal ganglia/watershed (BG/W) score. Infants with scores of 0–2 in any region were categorized as no/mild injury, and infants with scores greater than 3 in any region were coded as moderate/severe injury.

### Statistical analysis

Distributions of data values were first examined using descriptive statistics appropriate for measurement level to identify any missing or outlying values. A natural log transformation was applied to the heart rate values to better conform to statistical model assumptions. General linear mixed models (GLMM) were utilized to compare least square means between total variability, power band (HF, LF, and LF/HF ratio), within‐subjects effect, category (none/mild, moderate/severe), between‐subjects effect, temperature (warming, cooling), and male (male: no/yes) levels. A GLMM approach was utilized as GLMM analyses allow for missing data and can accommodate repeated measurement of study subjects under different conditions, incorporate time varying covariates, flexible covariance structures, and a variety of dependent variable distributions (including counts and dichotomous variables) (Vonesh 2012). Due to small cell sizes for the male by temperature and category by temperature interactions (smallest denominator degrees of freedom = 1), those interactions were not included in any of the models. Conformance to statistical model assumptions was evaluated using diagnostic plots produced by the MIXED procedure in SAS 9.4 and a natural log transform was applied to improve conformance to statistical model assumptions. The AICC fit criterion (Hurvich and Tsai 1989) was evaluated to compare models, including covariance structures; the model with the best (smallest) AICC was chosen as the final model for interpretation. Statistically significant interactions were followed up with simple main effects analyses. In light of the small sample, an unadjusted 5% significance level was employed to preserve power in this hypothesis‐generating pilot study. Because of the small cell sizes, a GLMM analysis was not possible for the cytokines. Instead, differences between injury groups on selected cytokines were explored at each time point using *t*‐tests.

## Results

### Patient demographics

Our study group consisted of 16 subjects (Table 1). The mean gestational age for the 16 subjects was 37.8 ± 2 weeks (mean ± standard deviation,) and the mean Apgar scores at 1, 5, and 10 min were 1.6, 3.8, and 5.7, respectively. For the entire study population, the mean lactate and base deficit (drawn between 0 and 6 h) were ‐10.5 ± 5.2 and −17.6 ± 7.2, respectively. Participants were 50% male. When neonates were separated into two study groups based on their MRI injury, the neonates with mild or no injury were similar to the neonates with moderate to severe injury in regards to gestational age, Apgar scores, pressor requirement, initial lactate levels, and base deficits (Table 2). Of the 16 subjects, 5 infants met criteria for moderate/severe injury and the remaining 11 infants met criteria for none/mild injury based on MRI findings. The groups were similar in distribution in regards to sex, 45% males in no/mild injury and 40% males in the moderate/severe group. Forty‐four percent of the infants had injury in the basal ganglia and 38% of the infants had injury in the white matter. Of the 25% of infants with injury in both the basal ganglia and the white matter, all were in the moderate/severe injury group (Table 3).

**Table 1:** Infant characteristics.

Subject	GA (weeks)	Sex	APGARscores	Cord gas pH	Initial pH	Initial base deficit	Initial lactate	Pressor support	Mechanical ventilation	Status at discharge	Complication
1	36	F	0, 5, 6	a	6.76 (v)	−29 (v)	11.7 (v)	No	Yes, Brief	Alive	Abruption CPR
2	38	F	1, 1, 2	a	6.80 (c)	−17 (c)	10.6 (c)	No	Yes	Alive	CPR Infantile spasms
3	37	M	1, 3, 4	6.40	6.70 (a)	−25 (a)	14.1 (a)	Yes	Yes	Deceased	Seizures Failed extubation
4	42	F	2, 4, 5	6.89	7.00^a^ (a)	−16^a^ (a)	8.1^a^ (a)	Yes	Yes	Alive	Fetal distress MAS/PPHN
5	37	F	2, 5, 7	a	7.17^a^ (a)	−13^a^ (a)	13.6^a^ (a)	Yes	Yes	Alive	Decreased fetal movement PPHN
6	37	F	1, 1, 2	a	7.14^a^ (a)	−11^a^ (a)	1.8^a^ (a)	Yes	Yes	Deceased	Failed extubation TSPAN 7 duplication
7	38	M	3, 6, 8	6.80	7.04^a^ (v)	−13^a^ (v)	14.4^a^ (v)	Yes	Yes	Alive	
8	35	M	2,2,6	7.17	6.99 (a)	−18 (a)	17.7 (a)	Yes	Yes	Alive	NRFHT AV canal defect
9	38	M	2, 5, 5	7.09	7.19 (a)	−14 (a)	11.9 (a)	No	Yes	Alive	Shoulder dystocia
10	35	M	1, 6, 7	6.91	7.27 (v)	−2 (v)	4.9 (v)	No	No	Alive	Fetal bradycardia
11	35	F	1, 2, 5		7.41 (a)	−1 (a)	2.5 (a)	Yes	Yes	Alive	Seizures
12	38	M	3, 5, 6	6.70	6.90 (a)	−27 (a)	15.5 (a)	No	Yes, brief	Alive	Abruption NAS
13	39	M	1, 3, 5	6.70	6.90 (a)	−26 (a)	14.3 (a)	Yes	Yes	Alive	Abruption
14	39	F	1, 2, 5	a	6.90 (a)	−24 (a)	1.4^a^ (a)	Yes	Yes	Alive	Uterine rupture
15	40	F	2, 3, 5	6.99	7.28^a^ (a)	−13^a^ (a)	14^a^ (a)	No	Yes	Alive	MAS Possible chorioamnionitis seizures
16	41	F	1, 3, 7	7.25	7.30 (a)	−14 (a)	12.1 (a)	No	No	Alive	Shoulder dystocia seizures

CPR, cardiopulmonary resuscitation; MAS, Meconium aspiration syndrome; PPHN, Persistent pulmonary hypertension of the newborn; Non‐reassuring fetal heart tracing.

a Initial data not available from transferring hospital, gas obtained at Shand's Teaching Hospital; a = arterial, v = venous, c = capillary).

**Table 2:** No/mild injury versus Moderate/severe injury mean ± standard deviations for demographic data.

	No/mild injury (*n* = 12)	Moderate/severe injury (*n* = 4)	*P*‐value
Gestational age	37.7 ± 2.2	38.0 ± 1.7	0.8
1 min APGAR	Median 1, IQR 1.4	Median 2, IQR 2	0.8
5 min APGAR	Median 3, IQR 3	Median 3, IQR 3.5	0.9
10 min APGAR	Median 5, IQR 1.6	Median 7, IQR 4.5	0.7
Lactate	10.2 ± 5.4	11.17 ± 5.3	0.7
Base deficits	−18.6 ± 7.8	−15.4 ± 5.5	0.4
Need for pressors	50%	80%	0.3
Need for ventilator support	90%	80%	1

**Table 3:** Infant MRI findings and subject grouping per the Barkovich scoring criteria.

Subject	BG/W	Basal ganglia	White matter	Subject group	Radiologist notes
1	0	0	0	No/mild	
2	1	2	0	No/mild	Significant brain volume loss. Abnormal T1 signal in the basal ganglia.
3	4	4	5	Moderate/severe	Global and severe injury
4	0	0	0	No/mild	
5	3	4	5	Moderate/severe	Extensive areas of restricted diffusion as well as foci of cortical hemorrhage.
6	0	0	2	Moderate/severe	Bilateral, anterior watershed injury
7	3	4	5	Moderate/severe	Extensive injury
8	0	0	1	No/mild	Left frontal lobe white matter focal infarct
9	1	2	0	No/mild	Abnormal T1 signal noted in the basal ganglia
10	0	0	0	No/mild	
11	0	0	0	No/mild	
12	0	0	0	No/mild	
13	0	0	0	No/mild	
14	0	0	0	No/mild	
15	1	2	0	No/mild	Restricted diffusion noted in right basal ganglia as well as thalamus
16	3	4	4	Moderate/severe	Restricted diffusion noted in right basal ganglia and thalamus as well as anterior and posterior watershed injury.

### Heart rate variability during hypothermia

**Figure 1:**
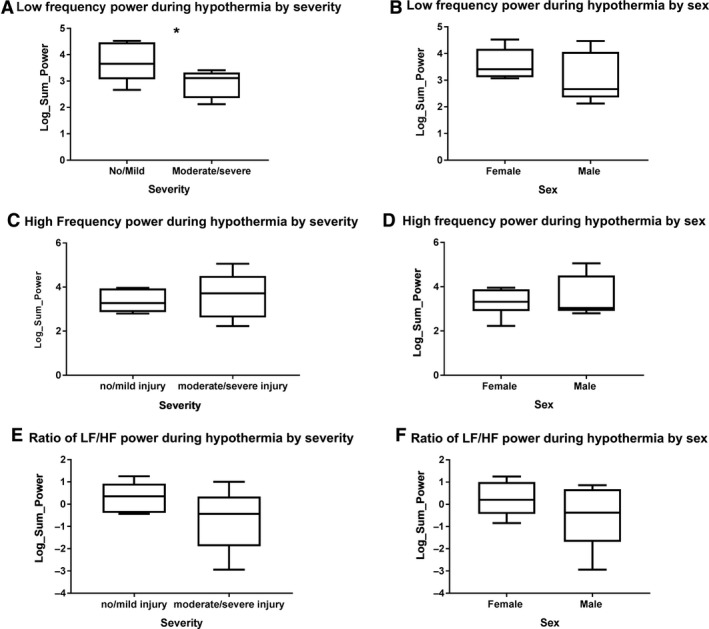
Heart rate variability trends during cooling. (A) Infants in the no/mild injury group have higher levels of low frequency (LF) power than infants in the moderate/severe injury group. (B) The LF power between males and females was similar. (C) The high frequency (HF) power was similar between no/mild and moderate/severe injury groups. (D) The HF power was similar between male and female subjects. (E) The ratio of LF/HF power was similar between the no/mild and moderate/severe injury groups. (F) The ratio of LF/HF power was similar between male and female subjects. *P < 0.05.

The MIXED procedure revealed that there was a trend (overall model: *F* (2,9) = 3.63; *P* = 0.052) for differences in LF power between no/mild and moderate/severe groups (main effect for injury group: *F* (1,9) = 5.51; *P* = 0.044), with infants in the no/mild injury group having higher levels of LF power than infants in the moderate/severe injury group (Fig. 1A). The LF power between males and females were similar (Fig. 1B). The HF power and LF/HF power were similar between the no/mild and moderate/severe injury groups (Fig. 1C and E). In addition, the HF power and the ratio of LF/HF power were similar between male and female subjects (Fig. 1D and F).

### Normothermic period heart rate variability

**Figure 2:**
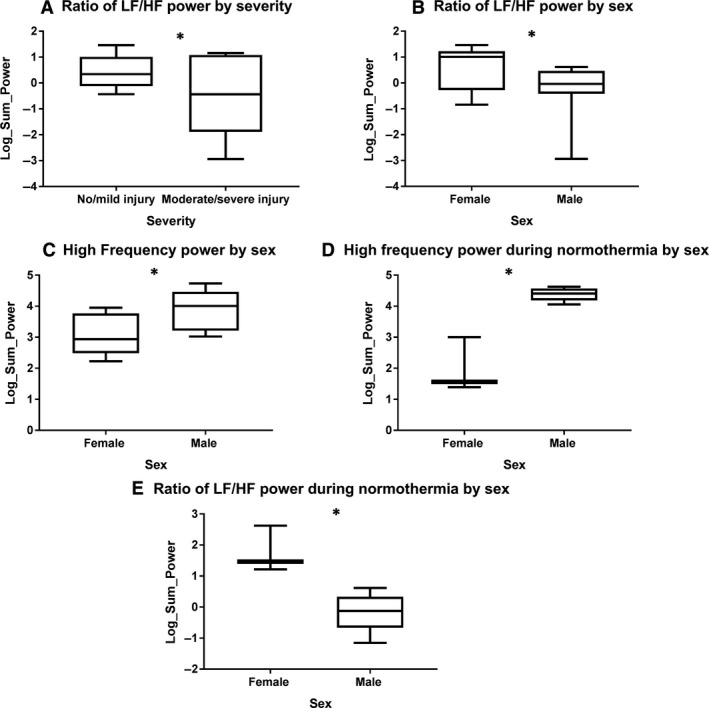
Significant heart rate variability trends trends over time or during normothermia. (A) The low frequency/ high frequency (LF/HF) ratio is higher in infants with less severe injury across the observation period. (B) The LF/HF ratio is higher in females across the observation period. (C) HF power is higher in males across the observation period. (D) HF power is higher in males during normothermia. (E) The LF/HF ratio is higher in females during normothermia. *P < 0.05.

There were no significant differences in LF, HF, or LF/HF powers during normothermia by severity. However, genders differed in the LF/HF ratio and HF power during normothermia. The HF power was higher for males compared to females (*P* = 0.0051, Fig. 2D). Further, males demonstrated a lower LF/HF ratio compared to their female counterparts during normothermia (*P* = 0.0008, Fig. 2E).

### Overall heart rate variability across time

The final GLMM model consisted of main effects for injury group, sex, temperature (hypothermia and normothermia) and frequency power as well as the interactions of frequency power and injury severity, frequency power and sex and frequency power and temperature. Given the small error degrees of freedom, both the temperature by frequency power interaction (*F* (2,6) = 0.46; *P* = 0.65) and the main effect for temperature (*F* (1,2) = 1.81; *P* = 0.31) were dropped from the initial model. Both frequency power by injury group (*F* (2,26) = 4.59; *P* = 0.02) and frequency power by sex (*F* (2,26) = 10.12; *P* < 0.001) were statistically significant, and followed up with simple main effects analyses.

Neonates with no/mild injury had a higher LSM for LF/HF ratio than those with moderate/severe injury across time (*P* = 0.01, Fig. 2A). Males had a lower LSM for LF/HF ratio over time than females (*P* = <0.001, Fig. 2B). Additionally, males had significantly higher least squares means (LSM) in regards to HF power over time than females (*P* = 0.02, Fig. 2C).

### Effect of mechanical ventilation on heart rate variability

Data for HRV in neonates requiring mechanical ventilation is shown in Table 4. There does appear to be some difference in HRV by severity when evaluating the LF power. Additionally, there appears to be a difference in HRV by severity, in particular the moderate/severe group, when assessing total frequencies. This finding is likely a function of the LF power component. However, the numbers in these groups were so small that no comparative inferential statistics were performed.

**Table 4:** Heart rate variability while on and off the ventilator for infants requiring mechanical ventilation.

Frequency	Category	Respiratory support	*N*	Mean ± SD	Median	Minimum	Maximum
LF	Moderate/severe	Off ventilator	1	35.64	35.6	35.6	35.6
		On ventilator	2	138.5 ± 110.1	138.5	60.7	216.4
	No/mild	Off ventilator	3	110.5 ± 84	158.3	13.5	159.6
		On ventilator	6	68.4 ± 38.5	63	33.4	132.7
HF	Moderate/severe	Off ventilator	1	44.4	44.4	44.4	44.4
		On ventilator	2	53.6 ± 4.0	53.6	50.8	56.5
	No/mild	Off ventilator	3	58.9 ± 53.7	38.7	18.3	119.7
		On ventilator	6	69.6 ± 54.2	55.7	18.3	160.3
Total Frequencies	Moderate/severe	Off ventilator	1	80	79.9	79.9	80
		On ventilator	2	192.2 ± 106	192.2	117.2	267.2
	No/mild	Off ventilator	3	169.4 ± 33.1	176.6	133.2	198.3
		On ventilator	6	138 ± 44	145.9	75.8	194.9
LF/HF ratio	Moderate/severe	Off ventilator	1	0.8	0.8	0.8	0.8
		On ventilator	2	2.7 ± 2.3	2.7	1.1	4.3
	No/mild	Off ventilator	3	4.3 ± 4.3	4.1	0.1	8.7
		On ventilator	6	2 ± 1.9	1.2	0.2	4.5

### Inflammatory biomarkers

**Figure 3:**
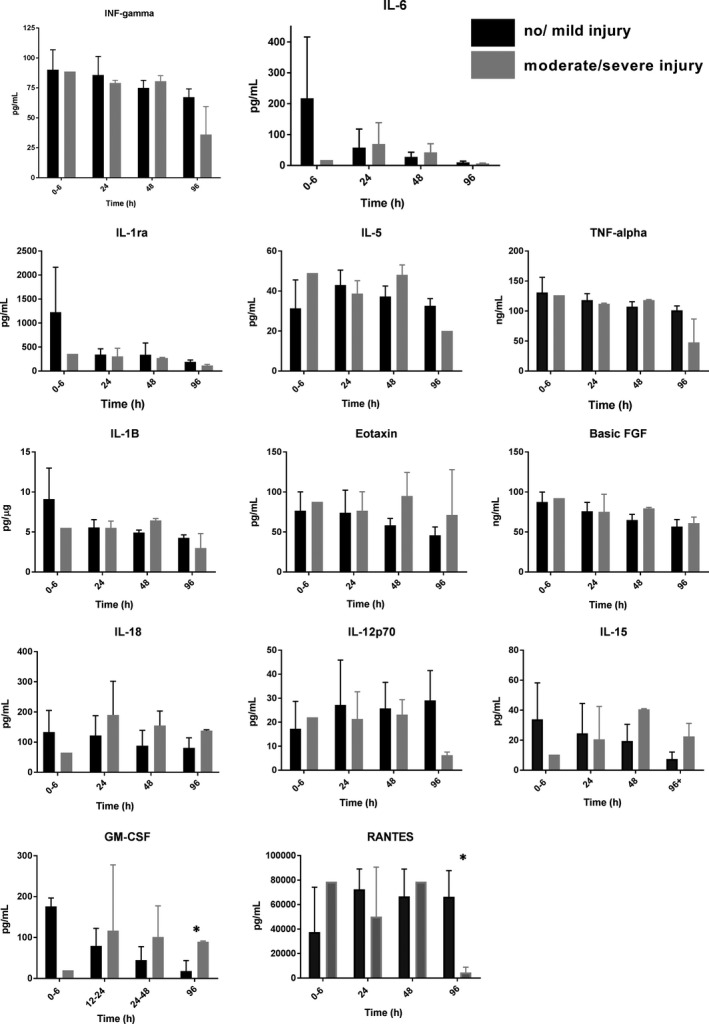
Biomarkers over time. *P < 0.05.

In the subjects that had serum in the biorepository (*n* = 10), inflammatory biomarkers were evaluated at each of the 4 time points (Fig. 3). Not all of the 10 subjects had enough serum samples available at every time point for analysis. No biomarkers were significantly different between infants with no/mild injury and those with moderate/severe injury during TH measured at 0–6, 24 and 48 h, though it would require a large effect size (Cohen's d) of 2.0 to provide 80% power. At 48 h, several biomarkers in the moderate/severe injury group demonstrated trends of increased levels including: IL‐1B (no/mild 4.93 ± 0.3 pg/mL vs. moderate/severe injury 6.45 ± 0.23 pg/mL; *P* = 0.06), IL‐5 (no/mild injury 37.25 ± 5.26 pg/mL, vs. moderate/severe injury 48.15 ± 4.93 pg/mL; *P* = 0.09), Eotaxin (no/mild injury 58.48 ± 8.47 pg/mL, vs. moderate/severe 95.06 ± 29.34 pg/mL; *P* = 0.06), basic FGF (no/mild 65.07 ± 7.05 pg/mL, vs. moderate/severe 79.57 ± 1.19 pg/mL; *P* = 0.06), IL‐15 (no/mild injury 19.45 ± 11.13 pg/mL, vs. moderate/severe is 40.65 ± 0.38 pg/mL; *P* = 0.06) and IL‐6 (no/mild 28.04 ± 14.87 pg/mL, vs. moderate/severe 42.30 ± 28.00 pg/mL; *P* = 0.50). At 96 h, the neonates with no/mild injury had significantly higher levels of RANTES (66422.90 ± 21367.80 pg/mL) compared to the neonates with moderate/severe injury (4536.0 ± 4307.70 pg/mL; *P* = 0.03). Also at 96 h, the no/mild group had significantly higher levels of GM‐CSF (17.83 ± 25.69 pg/mL) compared to the moderate/severe group (89.89 ± 1.26 pg/mL; *P* = 0.03). Biomarkers that demonstrated trends at 96 h include TNF‐alpha (no/mild injury 101.1 ± 94.24 pg/mL, vs. moderate/severe injury 47.96 ± 38.85 pg/mL; *P* = 0.06), IL‐12p70 (no/mild 29.11 ± 12.40 pg/mL, vs. moderate/severe 6.26 ± 1.29 pg/mL; *P* = 0.06), INF‐gamma (no/mild 67.19 ± 6.94 pg/mL, vs. moderate/severe 36.05 ± 23.41 pg/mL; *P* = 0.06), IL‐1ra (no/mild 192.3 ± 38.05 pg/mL, vs. moderate/severe 114.6 ± 24.67 pg/mL; *P* = 0.06), and IL‐18 (no/mild 81.26 ± 33.27 pg/mL, vs. moderate/severe 138.3 ± 2.81 pg/mL; *P* = 0.06).

## Discussion

This study suggests that neonates undergoing hypothermia with no/mild injury may exhibit higher LF power compared to neonates with moderate/severe injury. Several novel observations were made in this study. During therapeutic hypothermia, mechanical ventilation did not substantively impact HRV and rates of pressor use were similar between the two groups. In addition, this study indicated a difference in HRV between female and male neonates that has not previously been reported. After therapeutic hypothermia, males demonstrated a lower LF/HF ratio compared to females. The males' increased HF power likely accounts for the sex difference in the LF/HF ratio. Because this study was prospective, we were able to collect physiologic variables such as mechanical ventilation and analyze their effect. Finally, this study was able to explore 48 cytokines, many of which are novel and neither previously reported in neonates with HIE or previously reported in relation to MRI results.

The data in this study corroborates findings that improved outcomes are associated with higher LF power, particularly during cooling, and with a higher LF/HF ratio over the treatment period. In small samples of infants undergoing TH, time‐ and frequency‐based measures of HRV have differentiated between infants with subsequent neurological damage and infants with minimal or no neurological damage on MRI (Vergales et al. 2014; Goulding et al. 2017). In the earliest observations of HRV in infants with HIE, prior to the treatment with TH, infants with HIE had lower LF power and higher HF power than controls. The effect was more profound as the degree of HIE worsened, potentially indicating that HIE itself is associated with increased parasympathetic nervous system activity and decreased sympathetic activity (Aliefendioglu et al. 2012). Early animal models of hypoxia demonstrated early increases in LF, HF, and LF/HF acutely, but as rats acclimated to hypoxia, the sympathetic activity (LF power) decreased and the parasympathetic activity (HF power) increased (Kawaguchi et al. 2005). Children with brain injuries have also shown this physiologic response to injury. Children with elevated intracranial pressures and infants with low Glasgow coma scale scores had lower LF/HF ratios, whereas infants with favorable outcomes had higher LF/HF ratios (Biswas et al. 2000). In addition, lower LF power and lower LF/HF ratios were associated with higher mortality in cohorts of adults with sepsis (de Castilho et al. 2017). These associations are in contrast to work done in patients with rheumatoid arthritis (RA) and SLE. Trials in patients with RA and SLE indicated low HF power and higher LF/HF ratios while still showing lower LF power. These findings are felt to reflect the autonomic dysfunction found in autoimmune disease, though data correlating the degree of inflammation to HRV has been mixed (Aydemir et al. 2010; Adlan et al. 2014; Provan et al. 2017). We hypothesized that the HRV response in infants with HIE would mirror that of patients with autoimmune disease, given the significant inflammatory response. However, in studies of infants undergoing TH, the adverse outcome group had lower LF power and higher HF power over time compared to the favorable outcome group. In these infants, the best distinction between levels of neurological damage was detected approximately 24 h into the cooling treatment and after 80 h of life (Massaro et al. 2014). Recent studies have shown that HRV may predict brain injury pattern (Metzler et al. 2017), temperature itself causes changes in HRV (Massaro et al. 2017), and HF power was increased the most in infants with moderate EEG findings of HIE as compared to those with mild EEG findings of HIE (Goulding et al. 2017).

Given the findings of this study and evidence provided in previous studies, increased sympathetic activity during hypoxia, as evidenced by LF power or the ratio of LF/HF power, appears to be protective. Potentially, the sympathetic nervous system's role in maintaining adequate blood pressure and cerebral blood flow may potentiate the effects of ischemia. Alternatively, greater damage to the subcortical regions of the brain seen in more severe HIE may prevent activation of the sympathetic nervous system resulting in lower LF power. Studying the body's ability to manage the sympathetic nervous system during periods of ischemia may be paramount in designing future therapeutics for HIE. The novel findings of frequency‐based HRV that differ by sex will need to be accounted for in subsequent evaluations of HRV in infants with HIE. Additionally, while no comparative statistics could be performed, mechanical ventilation may be a confounding factor in particular groups of patients.

Studies of infant HRV in HIE have elucidated that frequency‐based measures of HRV may be related to inflammatory markers. Systemic inflammation‐modulating cytokine expression may be inversely related to HF power or parasympathetic control of HRV (Al‐Shargabi et al. 2017). Preliminary data indicates that levels of inflammatory biomarkers may help distinguish groups of neonates with poor outcomes from neonates with good outcomes after cooling. This study evaluated the largest group of inflammatory cytokines ever studied and included some cytokines that other studies have found to be associated with MRI severity, particularly IL‐6 and IL‐10. Additionally, this study was able to evaluate biomarker fluctuations over time. GFAP, IL‐6, IL‐8, IL‐10 and VEGF were previously found to be associated with outcome group (Chalak et al. 2014; Orrock et al. 2016). Interestingly, while most of these biomarkers were evaluated in our study (except GFAP), we did not observe such differences. IL‐6 and IL‐8 were qualitatively, but not statistically, higher at 0–6 h and lower at 24 and 48 h in the no/mild group compared to the moderate/severe group. IL‐10 levels were also higher at 0–6 h with persistence of the elevation at 24 h. Subsequently, IL‐10 was lower at 48 and 96 h in the no/mild group compared to the moderate/severe group. VEGF was inconsistent over time across groups. However, none of these biomarkers were significantly different at any time points.

We have identified a trend towards differences in RANTES and GM‐CSF by MRI injury severity. In infants with no/mild injury, GM‐CSF was lower at 96 h (*P* = 0.03), and RANTES was higher at 96 h (*P* = 0.03) than in infants with moderate/severe injury. These particular biomarkers were not previously implicated in the HIE pathophysiology. Both RANTES and GM‐CSF are pro‐inflammatory. GM‐CSF overexpression in many pathologic entities causes widespread inflammation. However, in general, infants with no/mild injury had an inflammatory profile that abated more quickly than in the infants with moderate/severe injury. This finding may indicate that some infants are better able to control this inflammatory response. RANTES, a chemokine that attracts many cells in the immune system and induces release of histamine from basophils and activates eosinophils, increased over time in infants with no/mild injury but remained stable in infants with moderate/severe injury. Thus, the induction of RANTES appears to confer a protective effect. In nearly all the biomarkers evaluated, including RANTES and GM‐CSF, infants could not be distinguished by outcome at the 0–6 h mark. Not unexpectedly, due to a widespread inflammatory response shortly after a hypoxic‐ischemic injury. As time passes from the insult, patterns of either upregulation or downregulation of various biomarkers occur in an effort to control this inflammatory response. Unfortunately, in this investigation, no singular pathway was identified.

This study was limited by small sample size. This limitation was especially salient in terms of unequal distribution of infants with no/mild injury as compared to those with moderate/severe injury. This study was further limited by the inability to secure data at each of the expected time points, in particular, for the inflammatory cytokines. This inability to obtain data collection was due to the complex clinical needs of these high acuity infants. While the observations of this study should be used cautiously, they further support HRV as a means to monitor infants, and they identify a need for further evaluation of these cytokines in future studies.

## Conclusion

While HRV has been previously studied in HIE, few studies have evaluated the effect of sex, pressor use, or mechanical ventilation on HRV in neonates undergoing TH (Ramaswamy et al. 2009; Fairchild and O'Shea 2010; Massaro et al. 2013; Vesoulis et al. 2017). While this study is limited by sample size, this adds to the body of literature that sex does appear to have an influence over HRV. It also identifies that mechanical ventilation might impact HRV for certain patients. Pressor use did not significantly differ between severity groups. This information allows HRV to be used as a marker of prognosis, regardless of the need for pressor support and indicates that future studies should consider controlling for mechanical ventilation. Finally, this study examined the largest number of inflammatory biomarkers to date and identified two novel biomarkers, RANTES and GM‐CSF, for further study. Our pilot data should be used to generate future studies to further evaluate these findings.

## Conflict of Interest

The authors of this study have no financial conflicts of interest. This body of work is original, has not been previously published, and has not been submitted for publication elsewhere while under consideration.
